# Medial Opening Wedge Osteotomy for Early Osteoarthritis of the Knee With Dr. Saigal's Plate: A Case Report With Review of Literature

**DOI:** 10.7759/cureus.74913

**Published:** 2024-12-01

**Authors:** Alok C Agrawal, Santu Sarkar, Harshal Sakale, Jayesh Rojasara, Ajit Saigal, Lohitesh S, Alok Rai

**Affiliations:** 1 Department of Orthopaedics, All India Institute of Medical Sciences, Raipur, Raipur, IND; 2 Department of Orthopaedics and Traumatology, All India Institute of Medical Sciences, Raipur, Raipur, IND; 3 Department of Orthopaedics, Mangalam Hospital, Varanasi, IND; 4 Department of Orthopedics, Joint Replacement and Reconstruction, All India Institute of Medical Sciences, Raipur, Raipur, IND

**Keywords:** dr saigal, hto, lateral hinge fracture, mechanical axis deviation, medial opening wedge osteotomy, medial open wedge osteotomy, miniaci, modified rust criteria, osteoarthritis (oa), western ontario and mcmaster universities osteoarthritis index

## Abstract

Knee pain in patients often involves varus deformity and unicompartmental osteoarthritis (OA). High tibial valgus osteotomy (HTO) is increasingly recognized as an effective treatment, as it realigns the knee's mechanical axis towards the healthier lateral compartment, delaying degenerative changes in the medial compartment and reducing the need for joint replacement.

This case report discusses two patients with bilateral knee arthritis and varus deformity who underwent medial opening-wedge high tibial osteotomy (MOWHTO) using Dr. Saigal's plate (Nebula Surgical Pvt. Ltd., Gujarat, India). The first patient, a 45-year-old male with a BMI of 29.3 kg/m², had a good range of motion (ROM) and no ligamentous laxity. The second patient, a 36-year-old female with a BMI of 30 kg/m², also exhibited good knee ROM and no ligamentous laxity.

Initial evaluation included comprehensive radiological assessments via four X-rays: anteroposterior (AP) view in 30-degree flexion, lateral view, skyline view for the patellofemoral joint, and a standing orthosonogram view from the hip to the toes. The surgical technique aimed to correct varus angulation with valgus overcorrection. Preoperative preparation followed the Miniaci Method, involving a weight-bearing AP orthoscan of the entire leg to determine the corrective angle.

Postoperatively, a protocol focused on fixation rigidity allowed toe-touch walking after six weeks. Suture removal occurred on the 14th day with no NSAIDs administered. Data were collected preoperatively, intraoperatively, and at three, six, and twelve months postoperatively. Primary outcomes included the Oxford Knee Score (OKS), Western Ontario and McMaster Universities Osteoarthritis Index (WOMAC), and ROM. Secondary measures assessed mechanical axis deviation (MAD), correction of varus angulation, pain levels, and complications using the Modified RUST criteria for osteotomy site evaluation.

At the final follow-up, both patients showed excellent clinical outcomes with pain-free joint motion and optimal limb alignment. No complications such as infection, hardware failure, or need for total knee replacement were reported. The mean preoperative OKS significantly improved, indicating the procedure's effectiveness in enhancing function and quality of life. The WOMAC pain and functional subscores also improved consistently over the year. Although there was a temporary decrease in knee ROM initially, it rebounded by the final assessments. Overall, the intervention was safe and successful, with no deep infections, deep vein thrombosis, lateral hinge fractures, varus collapse, or implant failures reported.

## Introduction

High tibial osteotomy (HTO) is a common treatment for individuals with varus knee deformity, regardless of the presence of uni-compartmental osteoarthritis (OA) of the knee. It is recognized as a joint-preserving procedure that can slow or stop the degenerative process [1]. HTO works by shifting the weight-bearing axis away from the center of the knee joint, reducing the load on the medial compartment and slowing degeneration in that area. Various surgical techniques for HTO have been documented, including the Ilizarov method, medial opening, lateral closing wedge, and dome osteotomies [2]. The most effective approach, however, is the Medial Wedge Opening HTO (MWOHTO) [3]. By repositioning the mechanical axis laterally, HTO creates conditions that promote cartilage regeneration in the medial compartment [1].

The medial wedge open osteotomy (MWOHTO) procedure has a lower risk of complications compared to lateral closing wedge osteotomy. These complications can include compartment syndrome, peroneal nerve injury, extensive lateral compartment muscle detachment, the need for proximal fibula osteotomy, and limb length discrepancies. MWOHTO also allows for intraoperative adjustments to the necessary correction. Various fixation methods for osteotomy are available, including long and short plates, as well as non-locking and locking plates and spacer plates. While a 4-hole short plate is easier to apply, it has been associated with higher rates of complications due to plate failure. MWOHTO can be performed with or without the use of bone grafts. Autologous bone grafts can be harvested and reshaped to fill the osteotomy site, or bone substitutes such as tricalcium phosphate granules, hydroxyapatite, or acrylic cement can be used [4]. Several studies indicate that the rate of bone union does not differ significantly in cases involving medial opening-wedge HTO [5]. However, the use of autologous bone grafting can prolong the procedure and increase morbidity, including persistent pain at the donor site, risk of infection, paresthesia, and a higher likelihood of requiring blood transfusions [6]. Instances of correction deficits and delayed bone union have been observed when locking plates or plates with bone grafts are not utilized [7]. As a result, many experts prefer using long plates with small metallic spacers to stabilize the osteotomy until union occurs. Both clinical and biomechanical studies have shown that longer locking plates exhibit superior resistance to compressive and rotational forces compared to shorter plates [4,8]. Although the Tomofix plate is recognized for its biomechanical advantages in high tibial osteotomy, its bulky design may lead to discomfort for Asian patients, with local pain being a common adverse effect following HTO with Tomofix plates [9]. In response to these concerns, a thinner, anatomically adapted plate has been specifically engineered for MWOHTO.

The Saigal plate (Nebula Surgical Pvt. Ltd. Gujarat, India) system utilizes a uniaxial locking fixation method based on the principles of locking compression plates. This system features locking holes that provide angular stability across the osteotomy, allowing screws to secure both the plate and the cortical bone. Importantly, the upper third of the plate is contoured to better match the medial condylar flare of the tibia, and its reduced thickness is designed to minimize irritation to surrounding soft tissues. A study by Zahra Hayatbakhsh et al. examined the impact of the Level of Fixation (LOF) on the performance of HTO plates. The findings indicated that increasing the bend of the plate improved LOF, enhancing the stiffness of the bone-implant construct and significantly reducing pressure across the lateral metaphyseal hinge (from 182 MPa to 71 MPa). However, higher LOF also resulted in increased stresses within the fixation construct, with stress rising from 187 MPa to 258 MPa [8]. The study determined that a bone-implant gap smaller than 2.3 mm is safe, with optimal biomechanical efficacy observed at a gap size of 0.5 mm [8]. Despite numerous comparative studies on the biomechanical properties of the TomoFix system and other HTO fixation systems, the critical role of anatomical fit (LOF) in plate performance has often been overlooked. Extensive finite element analysis has shown that gaps between the plate and bone significantly compromise structural stability, increasing the risk of failure and exposing the construct to excessive forces. This can lead to complications such as screw and plate fractures or loosening [10,11]. Dr. Saigal's plate maintains a bone-implant gap of less than 0.5 mm, yielding favorable clinical and radiological outcomes for MOWHTO [12]. It incorporates a medial rectangular block to enhance LOF, thereby reducing the likelihood of failure. Additionally, this system presents a cost-effective solution, making it especially suitable for the Indian context.

This case report includes one male and one female having bilateral disease with symptomatic knee pain on the medial side with varus deformity. They underwent MOWHTO procedures performed by the same surgeon utilizing Dr. Saigal's plate.

## Case presentation

Case 1

In case 1, the patient is a 56-year-old female presenting with bilateral unicompartmental osteoarthritis of the knees, characterized by a varus deformity of 20 degrees in the right knee and 15 degrees in the left knee preoperatively. Her body mass index (BMI) is 29.3 kg/m², which classifies her in the overweight category, potentially contributing to her knee pathology.

Clinically, the patient exhibits a commendable range of motion (ROM) in both knees, indicating preserved joint function despite the significant osteoarthritic changes. Notably, there is no evidence of ligamentous laxity, which suggests that the cruciate and collateral ligaments are intact and stable, minimizing the risk of intraoperative complications and facilitating a more predictable postoperative recovery.

The total varus mechanical axis (TVMA) deviation is 5 degrees, which is critical for planning corrective osteotomy to realign the mechanical loading through the knee joint. Preoperative assessment included the use of standardized knee scoring systems.

Imaging technique

**Figure 1:**
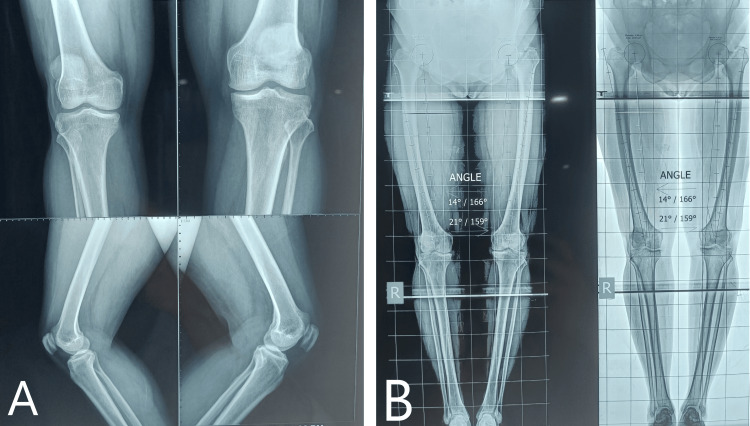
A) Pre-operative X-ray in weight bearing anteroposterior and lateral views. B) Orthoscannogram of both lower limbs in weight bearing standing position showing varus angulation.

**Figure 2:**
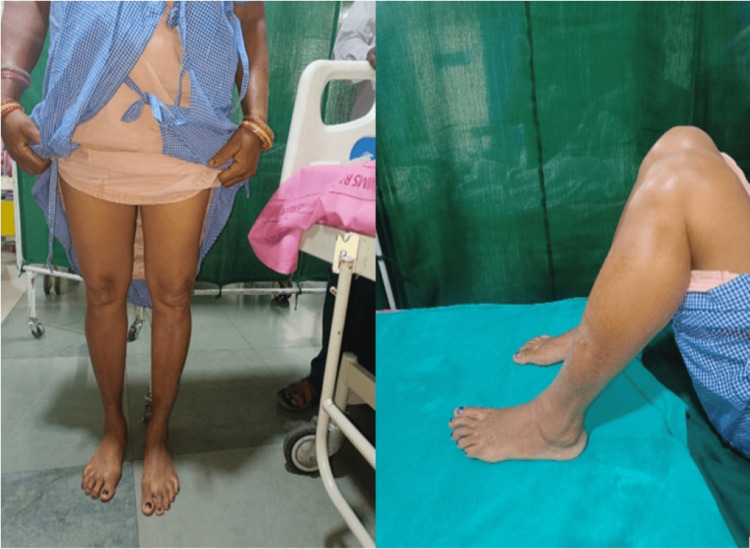
A) Pre-operative clinical images showing mild Varus deformity at both knee joints. B) Clinical image showing preoperative knee range of flexion.

For the initial assessment, four X-rays (Figure 1) are required: anteroposterior (AP) in 30-degree flexion, lateral, skyline view (axial view) for the patellofemoral joint, and a standing orthosonogram from the hip joint to the toe for both sides (Figure 2). If a physical examination shows ligament looseness, additional stress views should be taken.

Informed consent

Informed consent was obtained from all participants in the study.

Surgical technique

Preoperative Preparation

**Figure 3:**
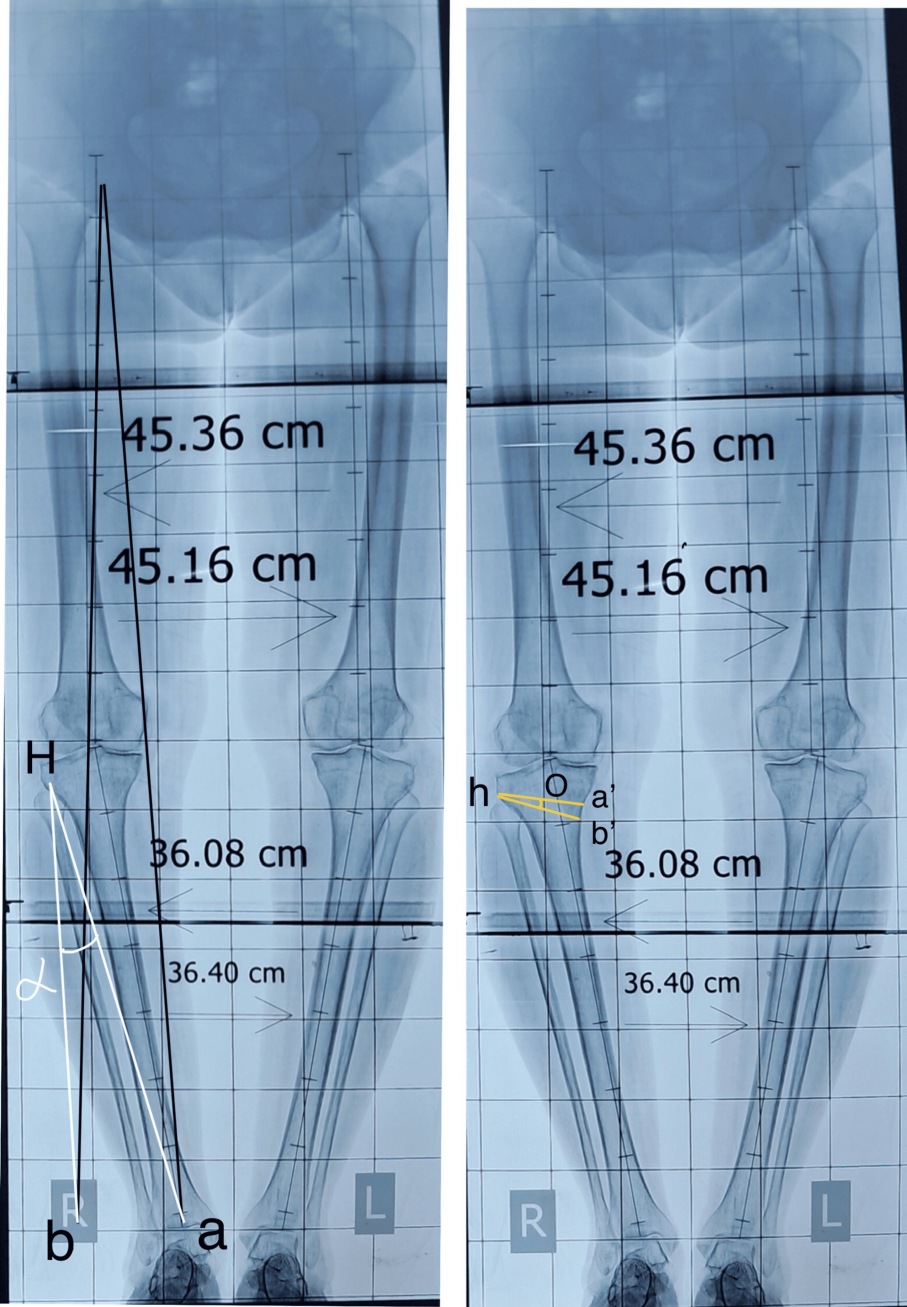
Illustrative diagram showing the calculation of the Alpha angle using the Miniaci method. A) To establish Mikulicz’s mechanical axis, draw a line from the femoral head to the ankle joint (α) and mark the new ankle center (b). Identify the hinge point (h) at the upper tibial metaphysis, then connect points (h) to (a) and rotate to intersect (b). Finally, connect (b) and (h) to form the angle α. B) This correction angle is translated at the medial osteotomy site 4 cm below the medial joint line extending up to lateral hinge.

**Figure 4:**
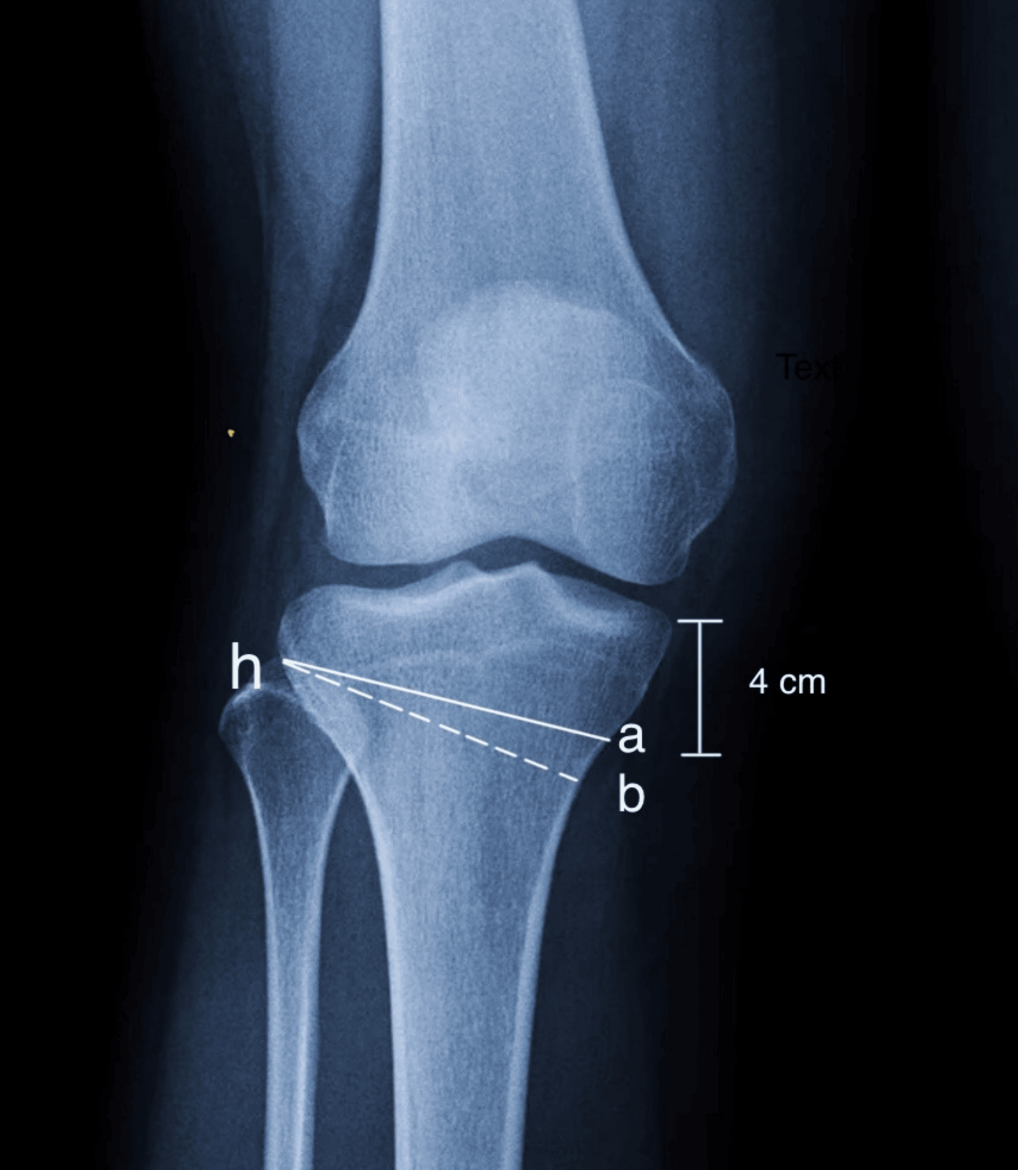
Identification of lateral hinge point. Hinge point is marked with K- Wires which is entered immediately superior to the pes-anserinus tendons. The osteotomy's hinge is located in the upper 1/3rd of the tibio-fibular joint—Medial Wedge Opening HTO surgical Technique

A thorough preoperative plan is crucial for the procedure's success. The Miniaci Method is recommended for this planning and should involve an orthoscannogram of the entire leg in a weight-bearing AP view [13]. Determine Mikulicz’s mechanical or weight-bearing axis of the lower limb by drawing a line from the center of the femoral head to the center of the ankle joint (a). After making any necessary corrections, mark the intended center of the ankle (b) and draw a new weight-bearing axis starting from the center of the hip to point (b). Identify the hinge point (h), typically located at the outer third of the upper tibial metaphysis, about 1.5 cm below the lateral joint line with the limb internally rotated at 30 degrees for optimal placement [14]. Connect point (h) to point (a) and adjust the line (h-a) in an arc to intersect point (b). Finally, connect (b) and (h). The angle formed between lines (h-a) and (h-b) is the opening angle (α). For the osteotomy at the level of the medial metaphysis, project angle (α) downwards to the medial tibial metaphysis, about 4 cm below the medial joint line, marking it as angle (o). The osteotomy height (a-b), which forms the base of the triangle with lines (h-a) and (h-b), is determined using Hernigou's trigonometric chart (Figures 3, 4).

Implant characteristics

**Figure 5:**
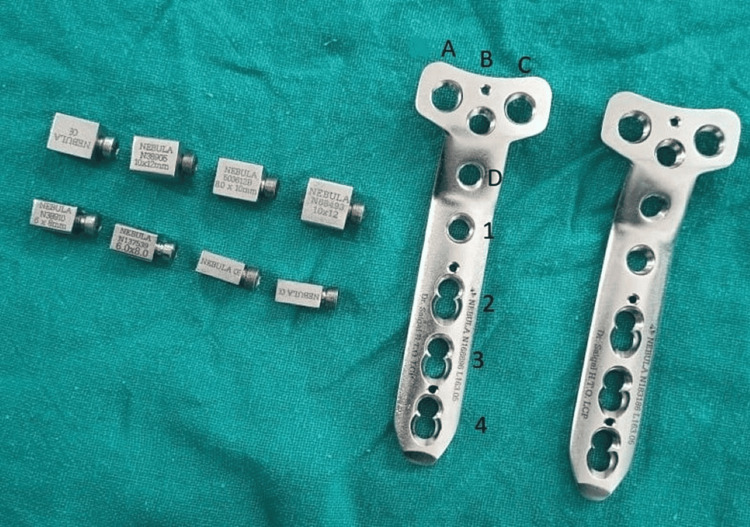
Implant characteristics of Saigal's plate.

The proximal part of the plate contains a horizontal row of holes from A to C. Hole D is present in the vertical limb above the bend. Hole 1 is present immediately below the bend. Holes A, B, C, D, and hole 1 are locking holes that allow locking screws of size 5 mm through it. Holes 2, 3, and 4 are combi-holes that can allow nonlocking or locking cortical screws of size 4.5 mm (Figure 5).

Approach

**Figure 6:**
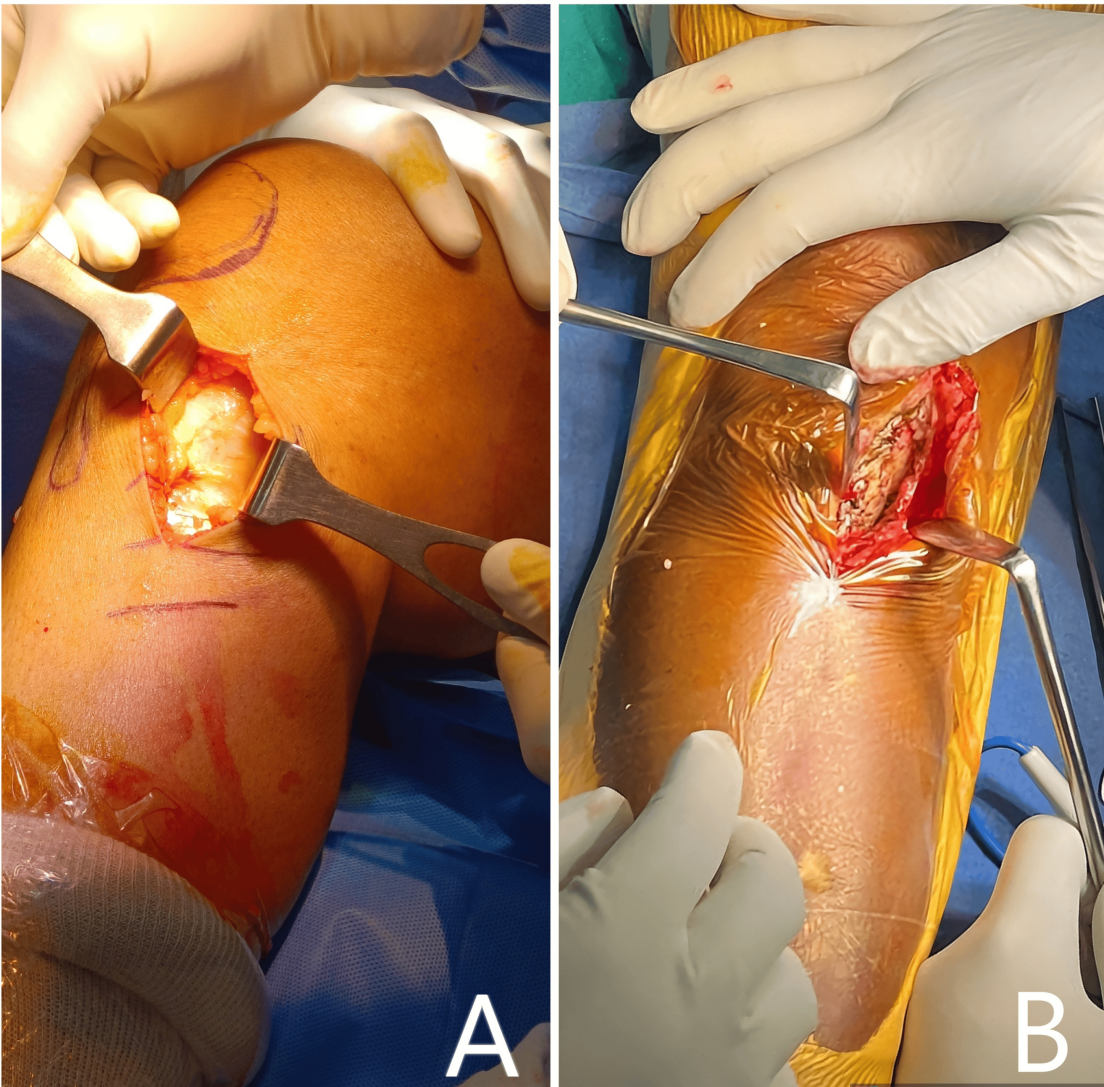
A) Minimally invasive approach to proximal medial tibia for MOWHTO. B) Subperiosteal dissection up to the proximal medial tibia.

To initiate the approach, position the leg in a fully extended manner. Proceed by marking the anatomic markers around the knee joint. Create an incision that is 6-8 cm in length over the anteromedial aspect of the proximal tibia. 1.5 cm inferior to the medial joint line and extending to the superior end of the pes anserinus tendons. Dissect and retract the sartorius fascia medially to expose the MCL. Finally, insert the Hohmann retractor into the infrapatellar bursa. With the help of a rasp, elevate the long fibers of the superficial MCL attached to the tibia until the posteromedial border of the tibia is visible; thereafter, another spiked retractor is placed beneath the posterior border of the tibia. During dissection, ensure that the saphenous nerve's dermal branches are not damaged (Figure 6).

Identification of the osteotomy site

**Figure 7:**
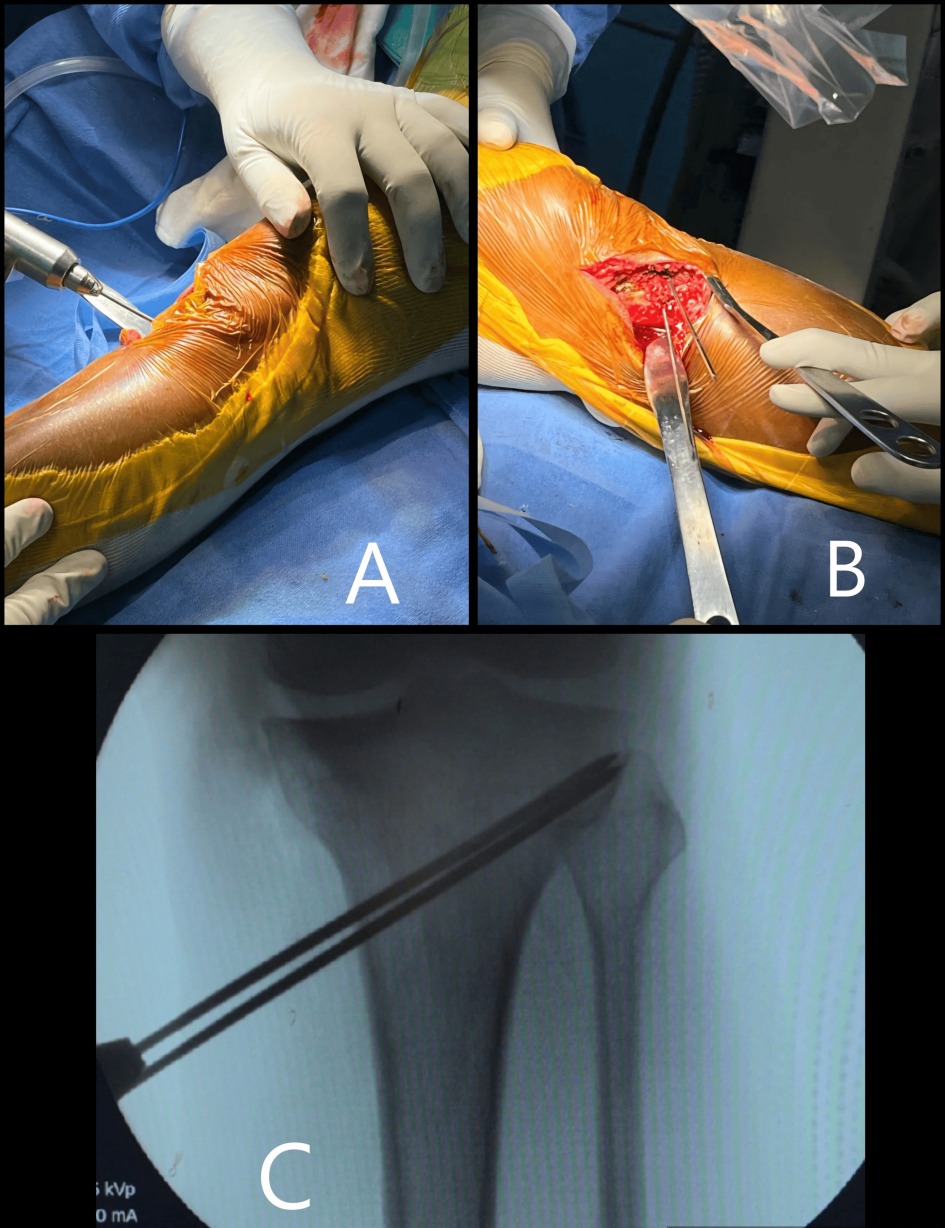
A, B) 2 Parallel K-wires are inserted aiming towards the lateral hinge point to mark the site of osteotomy. C) Identification and marking of the lateral hinge point using K wires.

**Figure 8:**
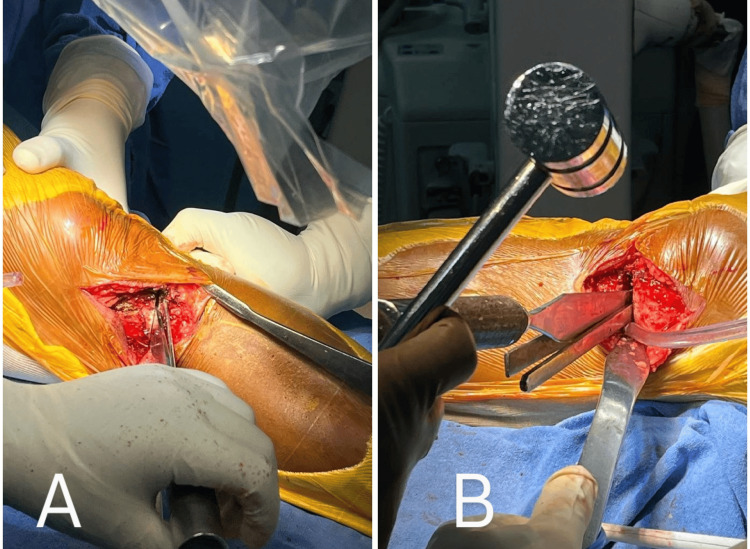
A) Horizontal osteotomy is done using the K wire as a guide. B) Spreading the osteotomy site using multiple chisels.

A safe zone was identified based on a cadaveric study conducted by Hans et al. [14] to determine the position for osteotomy. This safe zone is located at the level of the upper 1/3rd of the fibular head on the lateral 1/3rd of proximal tibial metaphysis. This safe zone is marked with 2 K-wires inserted approximately 4 cm inferior to the medial JL. The 1st wire is placed adjacent to the posteromedial tibial ridge on the superior boundary of the pes anserinus, while the 2nd wire is positioned 2.5 cm anteriorly and in line with the previous wire. To establish the depth at which to cut, place an additional wire of equal length over the k-wire entry site and assess the length difference. Using a thin oscillating saw, the osteotomy is started below and in line with the K-wires while ensuring that the outer 10 mm remains intact. Spread the medial 2/3 of the osteotomy utilizing three Lambotte chisels, or a calibrated lamina spreader may also be utilized. Safeguard the important soft tissue structures, including neurovascular bundles, located behind the posterior tibial cortex using a retractor. To preserve the tibial slope, k wires should be positioned relative to the slope of the tibia. This slope must be maintained after the osteotomy to prevent any change in the slope of the tibial plateau (Figures 7, 8).

Calculation of the amount of medial opening

The amount of medial opening is calculated using Hernigou's chart (Table 1) [11].

**Table 1:** Hernigou’s trigonometric chart for calculation of osteotomy width for MWOHTO. TomoFix Medial High Tibial Plate (MHT) for fixation of osteotomies of the Proximal Tibia

Mediolateral Diameter of the Osteotomy(mm)	Correction Angle (in degrees)																
		4	5	6	7	8	9	10	11	12	13	14	15	16	17	18	19
	50 mm	3	4	5	6	7	8	9	10	10	11	12	13	14	15	16	16
	55 mm	4	5	6	7	8	9	10	10	11	12	13	14	15	16	17	18
	60 mm	4	5	6	7	8	9	10	11	12	14	15	16	17	18	19	20
	65 mm	5	6	7	8	9	10	11	12	14	15	16	17	18	19	20	21
	70 mm	5	6	7	8	10	11	12	13	15	16	17	18	19	21	22	23
	75 mm	5	6	8	9	10	12	13	14	16	17	18	20	21	22	24	25
	80 mm	6	7	8	10	11	13	14	15	17	18	19	21	22	24	25	26

Plate positioning and fixation

**Figure 9:**
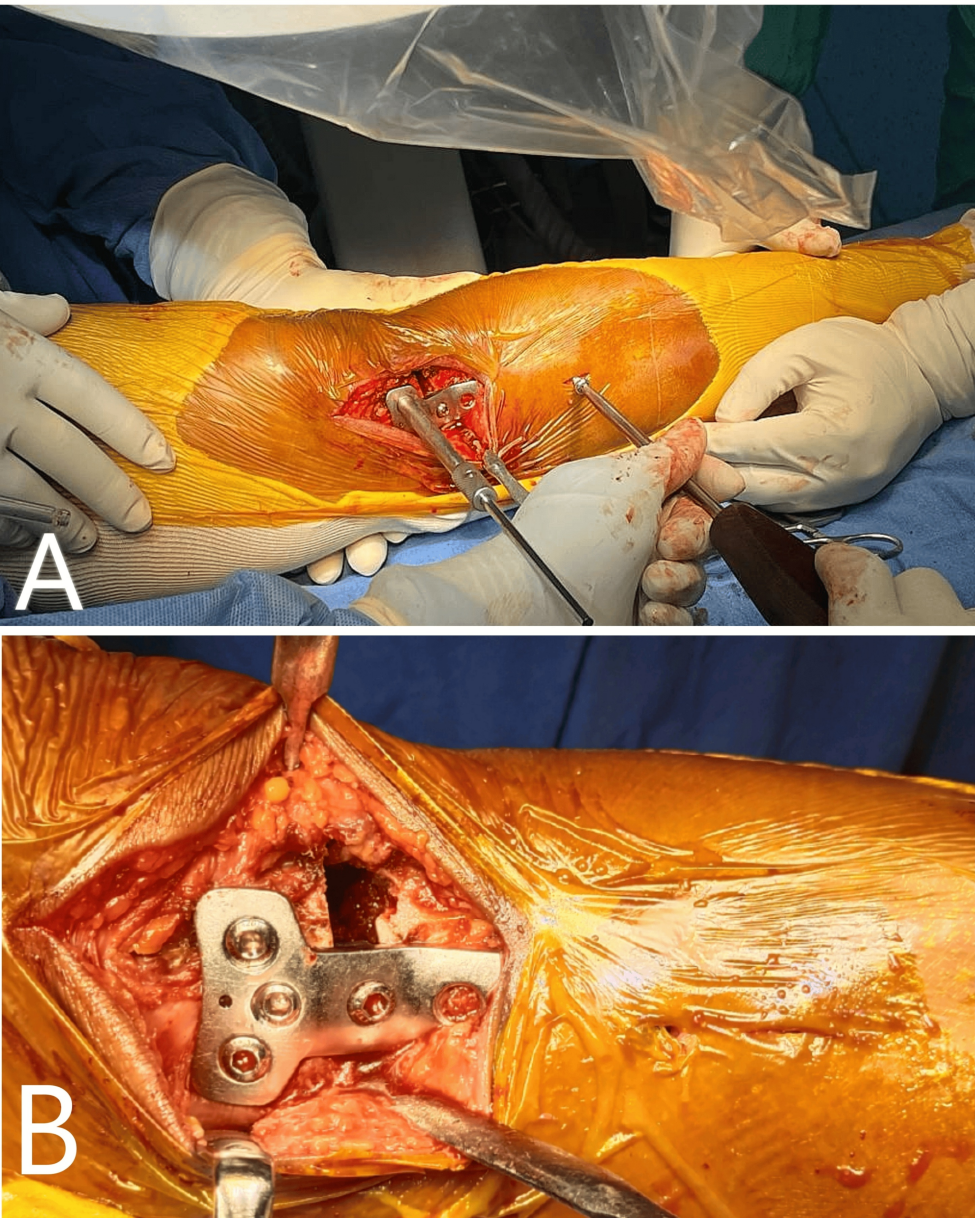
A, B) Fixation of Saigal's plate to protect the osteotomy site.

Lamina spreader forceps can be utilized to maintain the opening of the osteotomy site. After removing the guide wires, position the plate over the anteromedial surface of the proximal tibia. Align the shaft section with the tibial diaphysis and confirm with the help of an image intensifier. Insert the proximal locking screws approximately 1 cm below the joint line. To achieve secure fixation, a minimum of eight locking bolts, 4 proximal (A, B, C, D) and 4 distal (1,2,3,4) to the osteotomy site, are required. Begin by securing the proximal bone segment, followed by inserting a lag screw into the hole adjacent to the osteotomy, which secures the lateral hinge by providing compression across it. The remaining three bolts can be fixed using uni-cortical screws (Figure 9).

Bone healing after HTO

Healing begins at the lateral hinge and moves progressively in the medial direction. Three months following surgery, ossification and callus formation become apparent. Six months following the procedure, the new cancellous bone fills 75-80% of the space. In a year, full consolidation is seen in 90% of cases on radiographs [15].

Use of spacers In MOWHTO

To enhance the healing process and provide greater stability, many surgeons opt to utilize a bone graft or metallic spacers attached to the plate. Research has demonstrated that the inclusion of spacers can enhance the strength and expedite the healing of the bone at the defect. For patients who have increased chances of nonunion, such as smokers and individuals with high BMI, an autogenous bone graft may be useful in such conditions.

Postoperative rehabilitation

The fixation's rigidity is the primary determinant of the post-operative protocol. Patients undergoing plate fixation are allowed to start toe-touch walking as tolerated in the postoperative period for six weeks following surgery. Suture removal was done on the 14th postoperative day. NSAIDs were not administered as they could prevent bone healing.

Perioperative data collection

Data collection was done intraoperatively and postoperatively, including time of surgery, amount of blood loss and needs for transfusion, amount of correction needed, and occurrence of intraoperative complications. In the postoperative period, any persistent discharge from the wound, fever, DVT, and pulmonary embolism were taken into consideration.

Follow-up

All evaluations were done by a joint replacement fellow surgeon. Data collection was done preoperatively, intraoperatively, and postoperatively at three months, six months, and one year. For the primary outcome, parameters including Oxford Knee Score (OKS), WOMAC score, and ROM were recorded from index surgery and compared to preoperative scores. Secondary outcome criteria include measurement of TMVA, MAD (mechanical axis deviation), persistent or aggravation of joint pain, which required conversion into TKR, any incidence of implant failure, infection, or wound dehiscence, lateral hinge fracture, and proximal tibia fracture. To assess the union of the osteotomy site, the Modified RUST criteria (Radiological Union Score of Tibia) were utilized [15]. It involves evaluating 2 cortices (medial and lateral) on the AP view as well as the posterior cortex on the lateral view. A minimum score of 3 suggests that the fracture has not healed, while a maximum score of 7-9 indicates complete healing of the fracture. During the final follow-up, patients were asked whether the implant had been removed and the reason for its removal (Table 2).

**Table 2:** Radiological union score of tibia. Leow JM, Clement ND, Tawonsawatruk T, Simpson CJ, Simpson AH. The radiographic union scale in tibial (RUST) fractures: Reliability of the outcome measure at an independent centre. Bone Joint Res. 2016 Apr;5(4):116-21. doi: 10.1302/2046-3758.54.2000628. PMID: 27073210; PMCID: PMC5009237.

RUST (Radiological Union Score of Tibia) Score	Criteria
Score of 1	A visible fracture line without any sign of callus formation
Score of 2	A visible fracture line with some callus formation
score of 3	The fracture line is no longer visible with evidence of callus formation

**Figure 10:**
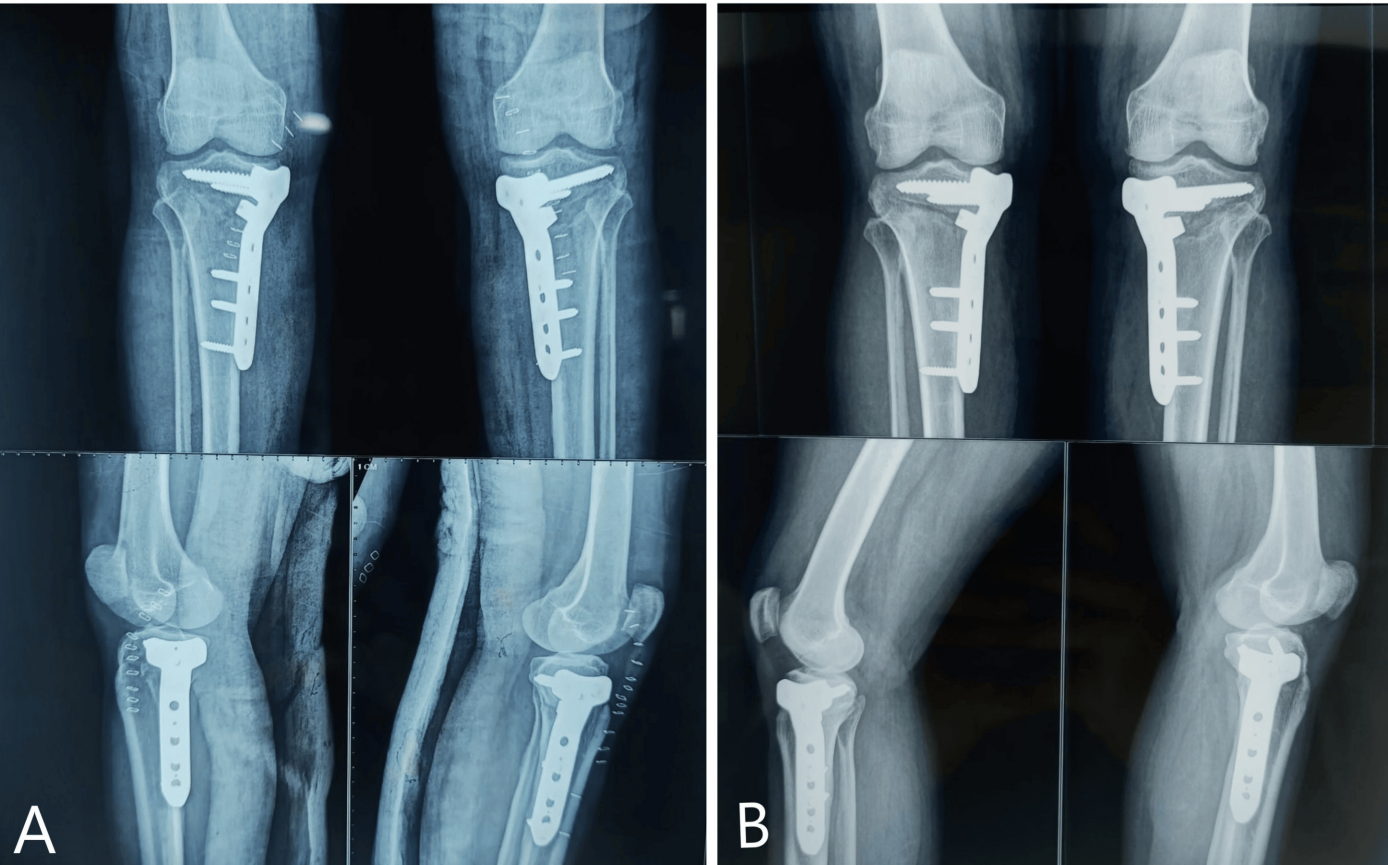
A) Immediate post-operative X-ray showing Implant in good position and the deformity was corrected to acceptable range. B) Three months follow-up X-ray showing remodeling at the osteotomy site.

In this case, the mean preoperative MAD was 17.6 mm of varus from the center of the knee joint. The patient was operated on with uniplanar bilateral high tibial osteotomy using Dr. Saigal's plate by minimally invasive technique. On POD2, the wound was healthy, and no signs of hematoma formation or any discharge were present. Passive, followed by active ROM, was started from POD2. The patient was discharged, and the hospital stay was uneventful. Sutures were removed on day 14, and wound healing was completed. Weight-bearing was started two weeks after the surgery (Figure 10, Tables 3, 4).

**Table 3:** Pre-operative and post-operative knee scores.

Knee Scores	Pre-operative score		Post-operative scores	
Oxford Knee score	Right Knee	Left knee	Right Knee	Left Knee
	21	19	47 at 1 year	48 at 1 year
WOMAC Score				
Pain	12	10	11 at 3 months	9 at 3 months
			8 at 6 months	7 at 6 months
			6.5 at 1 year	5 at 1 year
Stiffness	5	4	6 at 3 Months	4 at 3 months
			5 at 6 Months	2 at 6 months
			4 at 1 year	1 at 1 year
Functional	44	40	48 at 3 months	42 at 3 months
			42 at 6 months	35 at 6 months
			38 at 1 year	30 at 1 year

**Table 4:** Preoperative and postoperative knee alignment values.

Knee Alignment	Pre-operative value	value at 1 year of follow up
Varus angulation	14 degrees varus	2 degrees valgus
MAD	+17.06 mm	-6 mm

After three months of follow-up, the Oxford knee score improved from 21 recorded preoperatively to 43 at the final follow-up of one year. The WOMAC pain subscore was 12 during the preoperative period, which improved to 10 at three months, eight at six months, and finally to 6 at one year. WOMAC stiffness subscore initially increased from five recorded preoperatively to six at three months and then reduced to five at six months and then finally to four at one year of follow-up. WOMAC functional subscore taken preoperatively was 44, which initially increased to 48 at three months and then reduced to 42 at six months and finally to 38 at one year of follow-up. The knee ROM was initially reduced at the first follow-up and finally improved to full preoperative ROM at the final follow-up. The preoperative varus angulation was 14 degrees, which was corrected to neutral alignment with an overcorrection of 2 degrees. The MAD was 17.6 mm of varus preoperatively, which was corrected to 6.0 mm of valgus, which persisted at the final visit.

**Figure 11:**
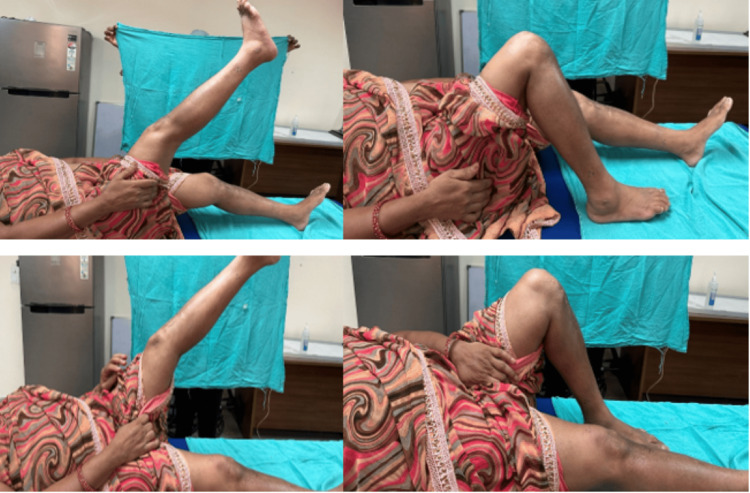
A) Complete extension of the right knee up to 0 degrees at one year of follow-up. B) Flexion more than 90 degrees in the right knee at one year of follow-up. C) Extension at left knee up to 0 degrees at one year of follow-up. D) Flexion more than 90 degrees in the left knee at one year of follow-up.

**Figure 12:**
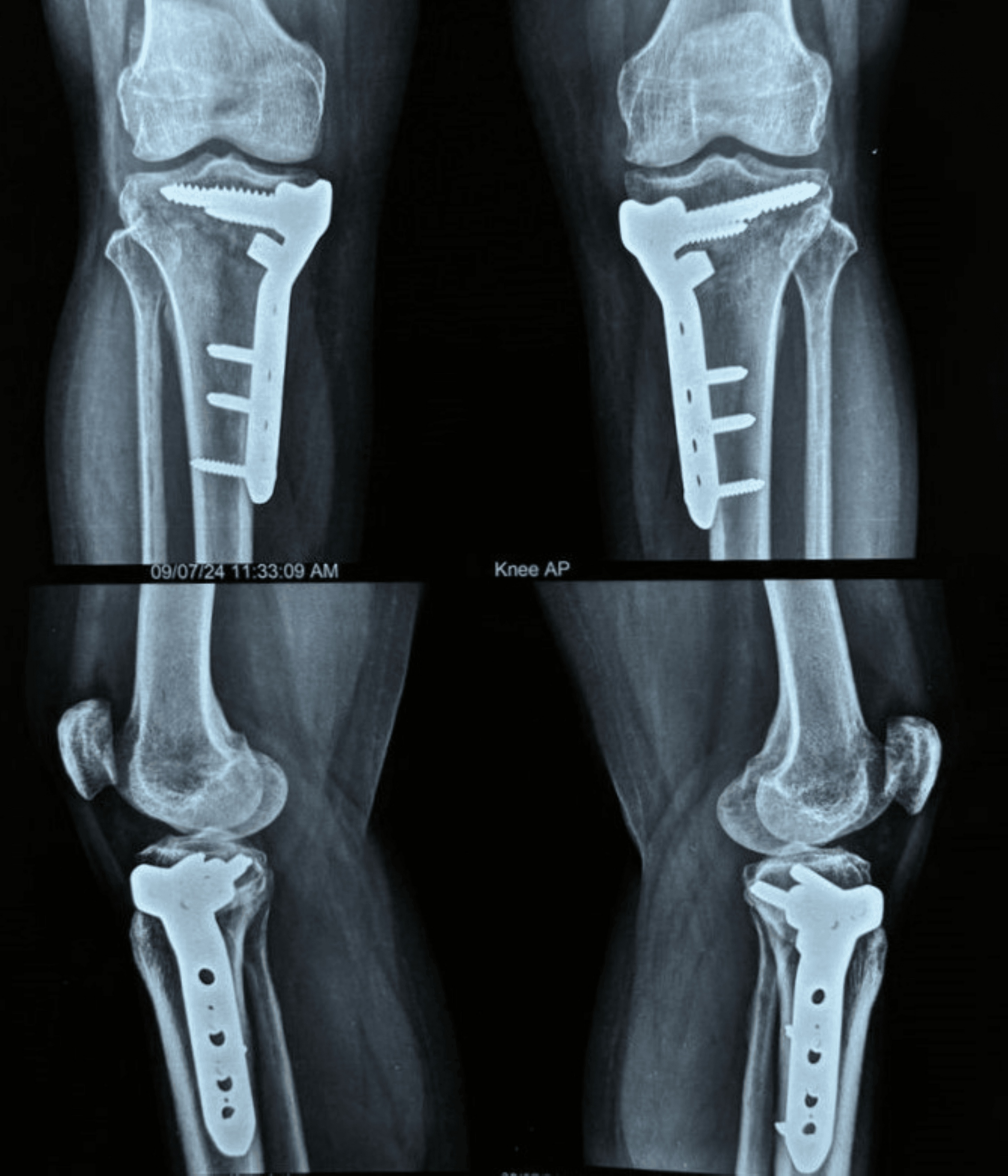
One-year follow-up X-ray showing healing of the osteotomy site with intact lateral hinge.

There was no incidence of wound dehiscence, DVT, lateral hinge fracture, or any other complication requiring implant removal or conversion to TKR. There was no incidence of nonunion of the osteotomy site. The average RUST score calculated one year postoperatively was seven, indicating good healing at the osteotomy site (Figures 11, 12).

Case 2

**Figure 13:**
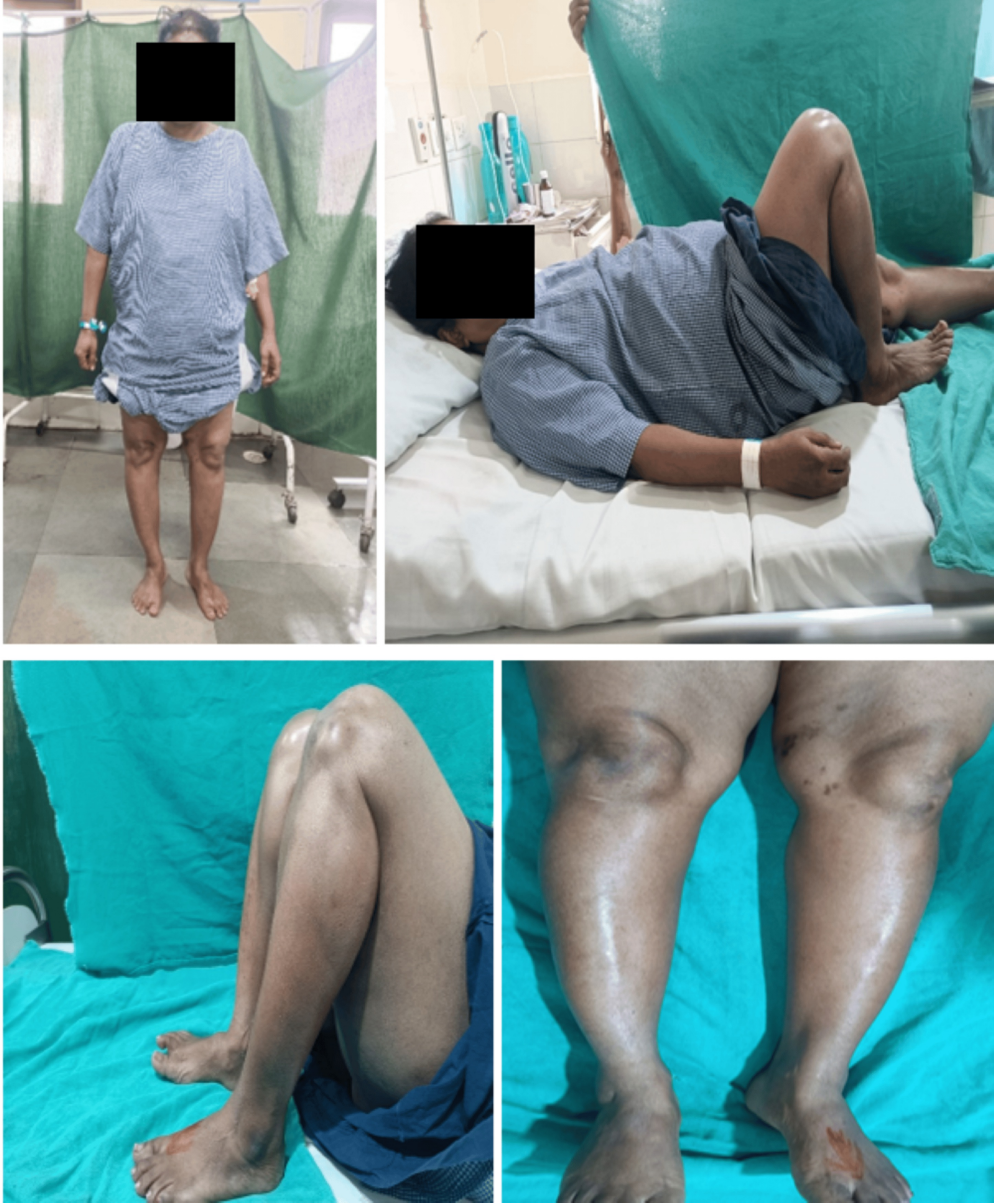
Preoperative clinical images. A) Standing weight-bearing front view. B) Maximum flexion of right knee. C) Maximum flexion of left knee joint. D) Lower limb alignment in supine position.

**Figure 14:**
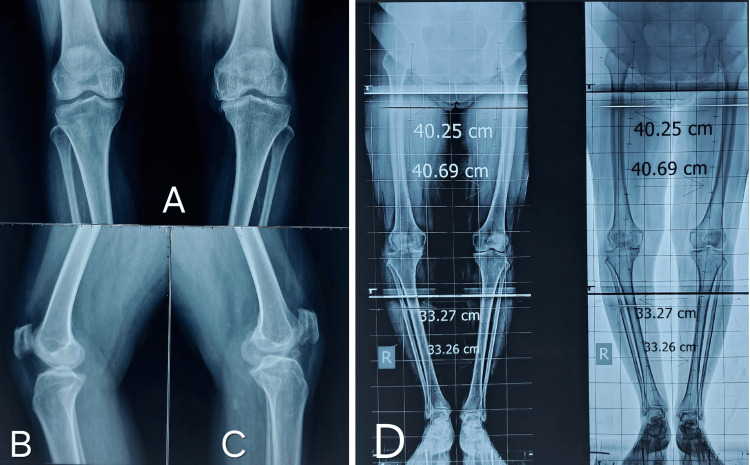
A) Radiograph showing anteroposterior view of the knee joint in standing weight bearing position. B, C) Radiograph showing lateral view of both knees. D) Standing orthoscannogram of both lower limb in weight bearing posture.

The clinical case involves a 50-year-old female with unilateral unicompartmental osteoarthritis in both knees, characterized by a 7-degree varus deformity in the right knee and a 12-degree deformity in the left knee. Her Body Mass Index (BMI) is 30.2 kg/m², indicating obesity, which may contribute to the progression of her osteoarthritis. Despite having a good knee range of motion and no ligamentous laxity, she presents with a tibial varus mechanical axis of 6 degrees. Additionally, she has no significant comorbidities, which is favorable for surgical intervention. Preoperative knee scores, which are referenced in Table 5, would provide insight into the severity of her condition and guide treatment recommendations (Figures 13, 14).

**Table 5:** Pre-operative and post-operative knee scores and alignment values for the right and left knee.

Knee Scores	Pre-operative score		Post-operative scores	
Oxford Knee score	Right Knee	Left knee	Right Knee	Left Knee
	18	26	47 at 1 year	48 at 1 year
WOMAC Score				
Pain	13	14	10 at 3 months	11 at 3 months
			6 at 6 months	8 at 6 months
			5 at 1 year	5 at 1 year
Stiffness	6	5	7 at 3 Months	6 at 3 months
			6 at 6 Months	5 at 6 months
			3 at 1 year	2 at 1 year
Function	46	42	49 at 3 months	45 at 3 months
			45 at 6 months	38 at 6 months
			34 at 1 year	30 at 1 year
Knee Alignment	Pre-operative Value		Post-operative Value	
	Right Knee	Left Knee	Right Knee	Left Knee
Varus Angulation	10 degrees	15 degrees	-4 degrees	-6 degrees
MAD	14 mm	17 mm	-5 mm	- 4mm

**Figure 15:**
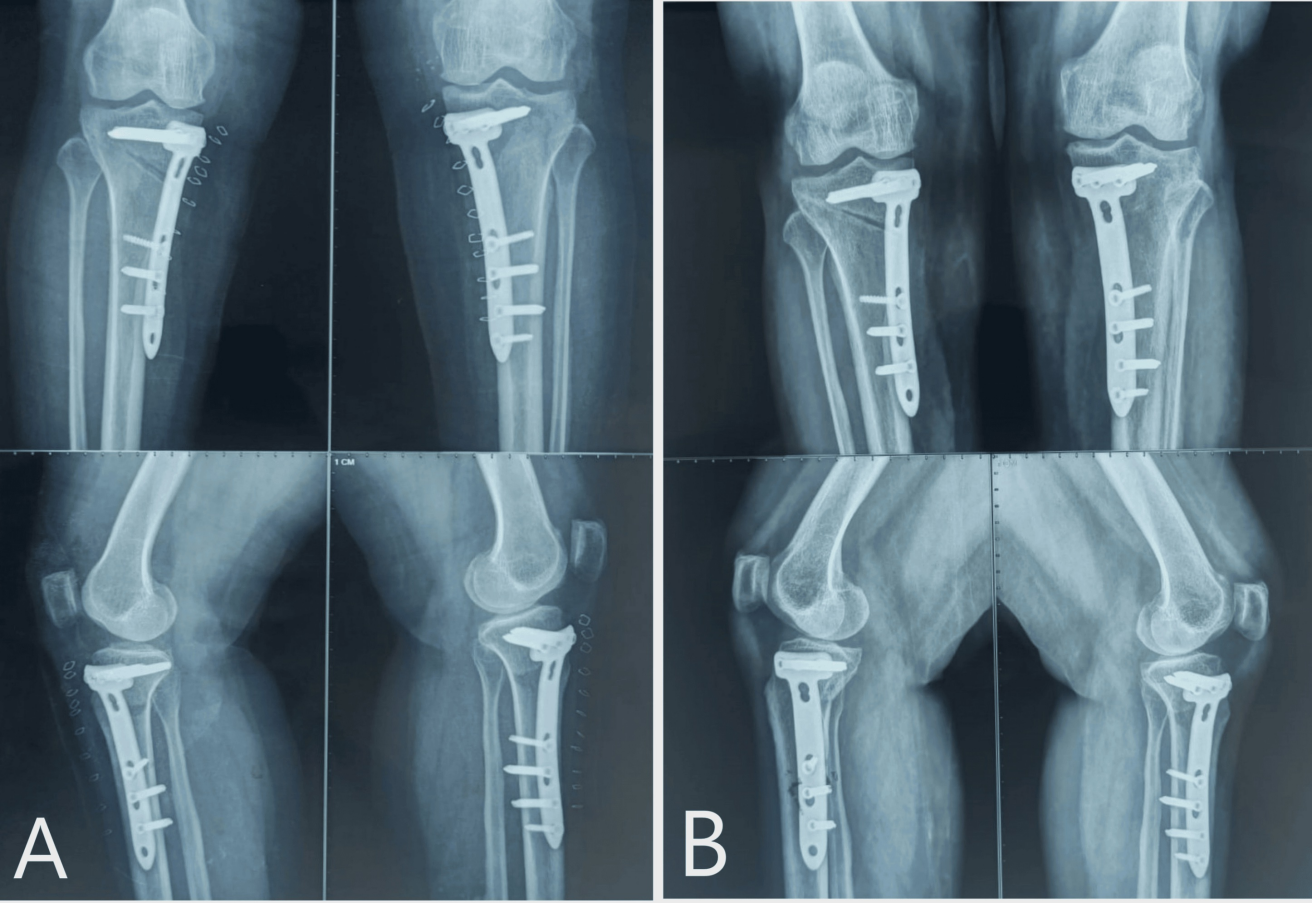
A) Immediate post-operative radiograph of MOWHTO using Dr Saigal's plate. B) Six months of follow-up X-ray showing progress of union in both knees.

The patient was operated on for bilateral high tibial osteotomy using Dr. Saigal's HTO plates and achieved deformity correction to neutral alignment with a slight overcorrection of 4 degrees. The patient started weight-bearing after one month (Figure 15).

Follow-up

After one year of follow-up, the Oxford knee score improved significantly from the preoperative value, and is shown in Table 6. WOMAC pain subscore improved significantly as compared to pre-operative values. The WOMAC pain subscore was 5 for both knees at one year of follow-up. WOMAC stiffness subscores initially increased from preoperative values for both of the knees at three months of follow-up, which finally reduced significantly at one year of follow-up. There was a similar decline in the WOMAC functional subscore at the initial follow-up, which finally improved for both of the knees at one year of follow-up. The knee ROM was initially reduced at the first follow-up and finally improved to full preoperative ROM at the final follow-up. The preoperative varus angulation was 10 degrees for the right knee and 15 degrees for the left knee, which was corrected to neutral alignment with an overcorrection of 4 degrees and 6 degrees of valgus for the respective knee. The MAD was 14 mm for the right knee and 17 mm for the left knee of varus preoperatively which was corrected to 5.0 mm and 4 mm of valgus for the respective knees, which persisted at the final visit.

**Figure 16:**
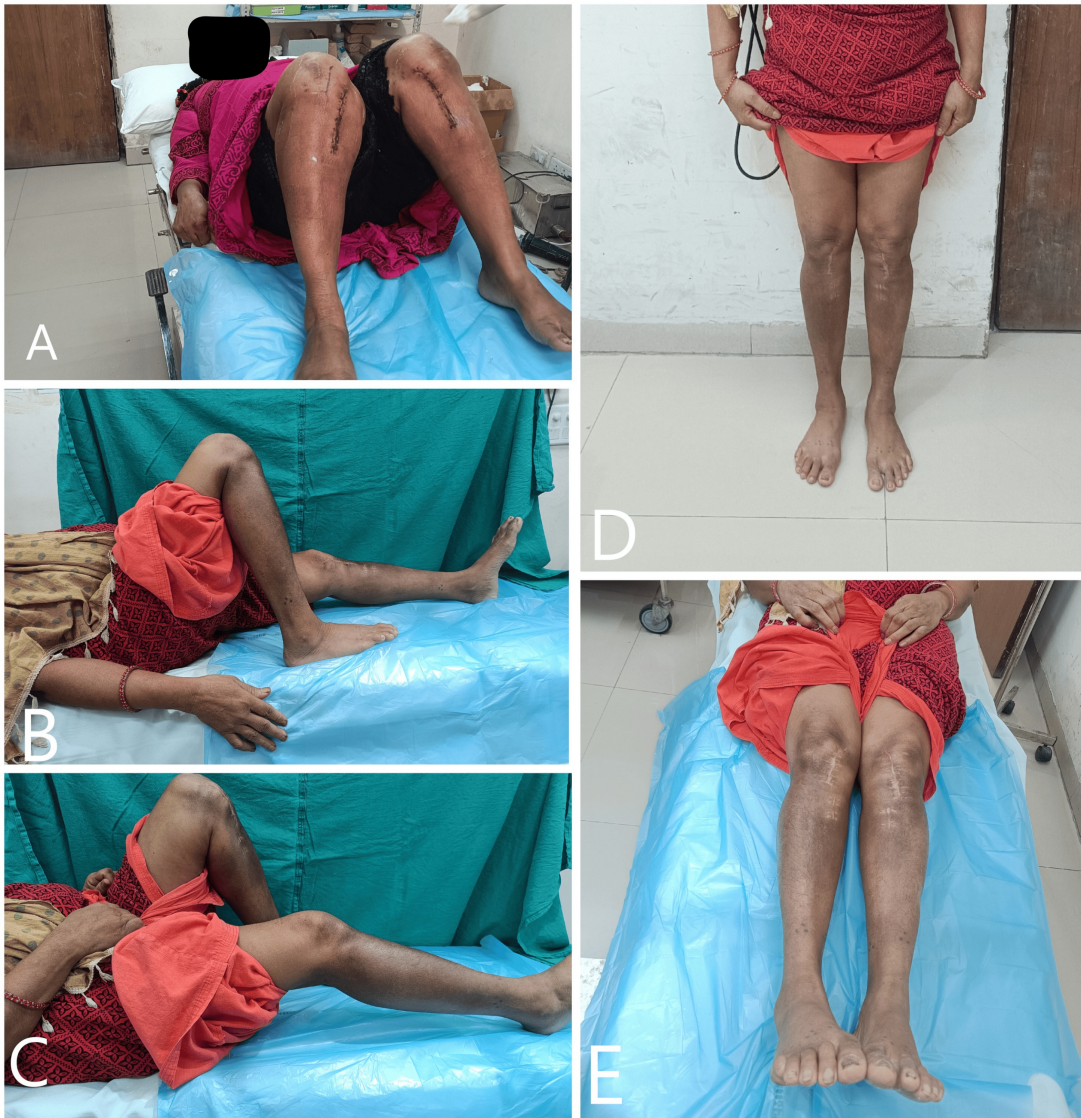
A) Knee flexion on immediate post-operative day 5. B, C) Knee flexion of Right and left knee respectively at one year of follow-up. D) Standing weight bearing clinical image at one-year of follow-up. E) Lower limb alignment at one-year of follow-up.

**Figure 17:**
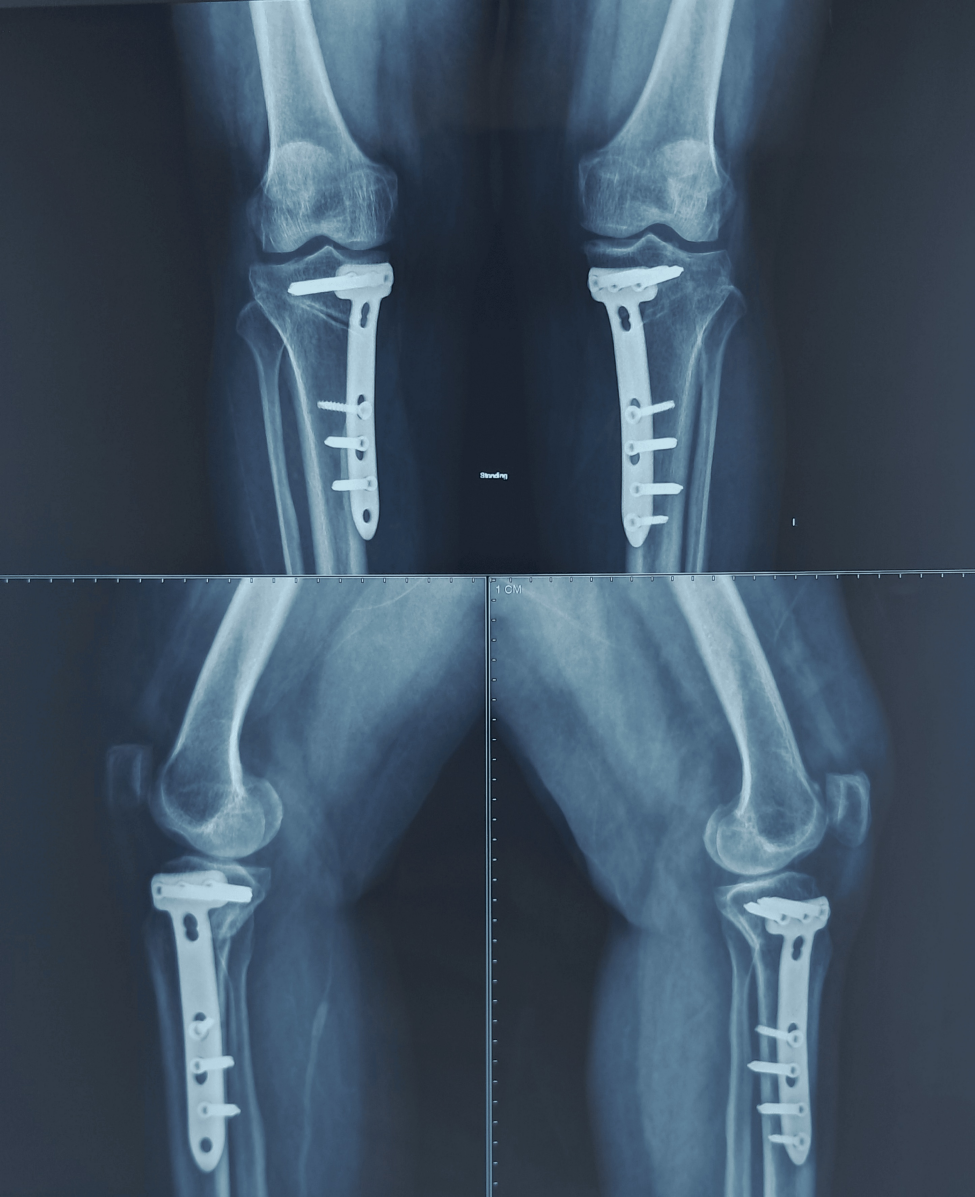
Anteroposterior and lateral radiographs at one year of follow-up showing union of all the cortices. The fracture line is visible only in the AP view of the right knee.

There was no incidence of wound dehiscence, DVT, lateral hinge fracture, or any other complication requiring implant removal or conversion to TKR. There was no incidence of nonunion of the osteotomy site. The average RUST score calculated one year postoperatively was eight, indicating good healing at the osteotomy site (Figures 16, 17).

Time for osteotomy healing

There was no incidence of nonunion of the osteotomy site. The average RUST score calculated six months postoperatively was seven for case 1 and eight for case 2.

Adverse effects

There was no incidence of superficial infection, hematoma formation, lateral hinge fracture, implant breakage, or proximal tibia fracture. There was no incident of deep infection, varus collapse, or implant failure that warranted implant removal. None of the patients required conversion to TKR for the persistence of pain.

## Discussion

Medial opening wedge osteotomy (MOWHTO) is an important joint-preserving surgery for medial unicompartmental osteoarthritis (OA) of the knee with varus deformity. This case report highlights the positive results of this surgical approach. Two patients underwent surgery using the modified Dr. Saigal's locking plate, which offers better biomechanics. At the final follow-up, both patients showed excellent outcomes, including pain-free joint motion and good limb alignment, with no complications such as infection or hardware failure that would require conversion to total knee replacement (TKR). The mean preoperative Oxford Knee score improved over one year, indicating enhanced knee function and quality of life. There was also a trend of improvement in the preoperative WOMAC pain and functional subscores, showing better knee pain and function. Although there was a temporary reduction in knee range of motion (ROM) initially, it returned to normal by the 12-month follow-up, reflecting successful joint function restoration. Notably, the mean preoperative varus angulation was corrected to a neutral alignment with maintained overcorrection. The absence of deep infection, deep vein thrombosis, lateral hinge fracture, varus collapse, or implant failure underscores the overall safety and success of the intervention.

In a previous investigation that employed a Tomofix plate for MOWHTO, lateral hinge fractures occurred in 22.6% of cases [16]. However, it was concluded that these fractures are not caused by the plate and do not impact the clinical outcomes of MOWHTO with locking plates. Keeping the lateral metaphyseal hinge intact during the osteotomy can significantly reduce the occurrence of lateral hinge fractures, which was implemented in this study. Overall, these findings support the efficacy, safety, and positive functional outcomes associated with the surgical approach in this study cohort. According to previous studies, the risk of nonunion in MOWHTO does not exceed that of close wedge osteotomy [17]. Complications related to the healing of the osteotomy site, like nonunion and implant failure, were not seen in this study, supported by the RUST score approaching near maximum. These findings support the conclusion that locking plates are ideal implants for MOWHTO, providing excellent clinical outcomes in younger patients with high physical demands [18].

Limitations of the study

The limitations of this study include a small sample size, which restricts the generalizability of the findings and may lead to potential bias. Additionally, the absence of a control group limits the ability to draw firm conclusions about the effectiveness of the intervention, as there is no comparator to evaluate against. Furthermore, the lack of randomization introduces the possibility of confounding variables influencing the results, making it difficult to determine causality. Future research should involve a larger study population with a randomized controlled design to enhance the reliability and applicability of the results.

## Conclusions

In summary, the Saigal plate system has the potential to be a noteworthy advancement in orthopedic fixation techniques. Its uniaxial locking mechanism not only enhances anatomical alignment but also minimizes irritation to adjacent soft tissues. The system's emphasis on maintaining tight bone-implant gaps contributes to improved stability and a reduction in complication rates, particularly for medial opening wedge high tibial osteotomy.

Furthermore, its efficacy in addressing bilateral medial knee pain and varus deformities highlights its role as a cost-effective solution, especially within the Indian healthcare context. Overall, the Saigal plate system offers an exceptional alternative fixation device for surgeons performing the MWOHTO procedure, ultimately promoting enhanced patient recovery and satisfaction. Further studies are needed to fully evaluate its efficacy and long-term outcomes.
